# Personalized prediction of adverse heart and kidney events using baseline and longitudinal data from SPRINT and ACCORD

**DOI:** 10.1371/journal.pone.0219728

**Published:** 2019-08-08

**Authors:** Gal Dinstag, David Amar, Erik Ingelsson, Euan Ashley, Ron Shamir

**Affiliations:** 1 Blavatnik School of Computer Science, Tel-Aviv University, Tel Aviv, Israel; 2 Division of Cardiovascular Medicine, Stanford University School of Medicine, Stanford, California, United States of America; 3 Stanford Cardiovascular Institute, Stanford University, Stanford, California, United States of America; International University of Health and Welfare, School of Medicine, JAPAN

## Abstract

**Background:**

The 2017 guidelines of the American College of Cardiology and the American Heart Association propose substantial changes to hypertension management. The guidelines lower the blood pressure threshold defining hypertension and promote more aggressive treatments. Thus, more individuals are now classified as hypertensive and as a result, medication usage may become more extensive. An inevitable byproduct of greater medication use is higher incidence of adverse effects. Here, we examined these issues by developing models that predict both cardiovascular events and other adverse events based on the treatment chosen and other patient’s data.

**Methods and results:**

We used data from the SPRINT trial to produce patient-specific predictions of the risks for adverse cardiovascular or kidney outcomes. Unlike prior models, we used both the baseline characteristics collected upon recruitment and the longitudinal data during the follow-up. Importantly, our cardiovascular predictor outperformed extant models on SPRINT participants, achieving AUC = 0.765, and was validated with good performance in an independent cohort of the ACCORD trial.

**Conclusions:**

Our study illustrates the importance of including longitudinal data for assessing personalized risk and provides means for recommending personalized treatment decisions.

## Introduction

The hypertension treatment guidelines published in 2017 redefine thresholds for aggressive treatment of patients with hypertension and high risk of cardiovascular (CV) events [1]. An inevitable consequence of greater pharmacotherapy is an increase in adverse events [2]. The new guidelines are based on the conclusions of the SPRINT study [3], a large clinical trial aimed at determining the benefit of aggressive vs. standard treatment for lowering the risk of CV events. The design and recommendations raise important questions regarding personalized treatments. To what extent can we predict CV events by using multiple clinical parameters available upon recruitment? Can longitudinal data improve such predictions? Can we predict adverse events that may arise from aggressive treatment? A possible starting point for answering these questions is the development of predictive models that can distinguish between patients more likely to benefit from the aggressive treatment and those who are at higher risk to experience adverse events.

The SPRINT study data can be utilized to address these questions. SPRINT measured the effect of intensive vs. standard treatment arms for reduction in systolic blood pressure (SBP) in 9,361 non-diabetic patients with high risk of CV events. Comparing the response of the two arms, the study showed a significant decrease in the number of CV events in the intensive treatment group, along with an increased risk of some adverse events, most prominent of which were kidney-related outcomes. These data were subsequently released as part of the SPRINT Challenge [4]; and several studies utilized it, some in combination with other data, to address questions related to personalized prognosis [5–7]. In particular, Patel et al. [8] and Basu et al. [9] presented predictors for CV and adverse events using logistic regression and Cox proportional hazards regression (CoxPH) [10], respectively, using only the data collected upon participant entry to the study.

The SPRINT data is of two types: characteristics taken from each patient on the day of enrollment (*baseline* data) and follow-up blood pressure measurements taken at periodic visits (*longitudinal* data). Here, we used the SPRINT data to provide novel predictive models combining baseline and longitudinal data and examined whether they outperform extant methods. Using supervised methods, we compared multivariable models that predict the risk of CV outcomes and kidney-related events based on baseline data only, or utilizing all data (baseline and longitudinal). Understanding the value of longitudinal data to prediction models is timely, as use of such data will rapidly increase in the near future owing to digital health appliances. Such appliances allow patients to measure their blood pressure at home [11], making time-series data available to the physician at the point of treatment decision and even on the first visit.

## Methods

The SPRINT data set has three components for each patient: baseline characteristics collected at the day of enrollment, longitudinal measurements taken over time, and clinical outcomes. There are 20 baseline parameters, describing demographic and medical information. The longitudinal data are systolic and diastolic blood pressure (BP) values measured at periodic visits (every 3 months) for each subject (mean number of visits per patient: 14.46, SD: 4.13. 135,205 visits in total). We used these time series to extract summary statistics for each subject. Using summary statistics of longitudinal data to characterize follow-up response is a well-established tool for medical applications [10]. S1 Table shows of the baseline and longitudinal features and their statistics in both study arms; the main SPRINT study outcomes are summarized in S2 Table. Here, we focused on two main outcomes: (1) the study’s primary outcome, namely CV events (myocardial infarction, acute coronary syndrome, stroke, heart failure and death from CV causes) and (2) kidney-related outcomes (novel Chronic Kidney Disease (CKD) for non-CKD patients at baseline, and Acute Kidney Injury or renal failure (AKI)). Patients with CKD at baseline were excluded from our prediction of novel CKD. Unlike previous methods that predicted all Serious Adverse Events (SAEs), we focused on the prediction of kidney-related outcomes only. Since kidney-related events were the most statistically significant adverse events in the SPRINT study, we reasoned that controlling those was most critical for personalized prognosis.

We constructed two prediction models. The *baseline model* utilizes as candidate predictors for model construction all baseline features (see S1 Table). Two constructed variables combining two baseline features, the ratio of HDL to total cholesterol and the ratio of eGFR to serum creatinine, were added based on prior knowledge. The *longitudinal model* uses as additional candidate predictors summary statistics extracted from longitudinal measurements as follows. For each visit, we computed the pulse pressure (PP, namely SBP minus DBP) and then summarized the series of PP values as the following features: the difference between the maximum and minimum, the mean, the slope of a linearly fitted curve, its R squared statistic and its F statistic. A linear curve is the simplest representation of the BP trend and is less prone to overfitting than higher rank curves. The R and F statistics reflect the strength of the trend. To avoid information leakage in cases where a BP measurement was taken close to the event, we followed a retrospective study design: we excluded measurements from the last **t** months (using **t** = 6 or 12) prior to the event or to the end of follow-up (S1 Fig). Patients with less than three measurements up to **t** months prior to the event/censoring were excluded from the longitudinal model (n = 435 (4.6%) and 634 (6.7%) for **t** = 6 and **t** = 12, respectively). All our predictors are multivariable, i.e., they use multiple features for prediction. In contrast, the SPRINT study is akin to a predictor that uses a single parameter (group assignment at recruitment) to predict the outcome.

For each individual, we used Cox proportional hazard regression on the baseline data to predict the risk for an outcome, and Logistic Regression on baseline and longitudinal data to predict the probability of the outcome. Final models were derived using lasso regularization [12,13] to account for collinearity between candidate predictors and to get compact, interpretable models. To evaluate the predictions, we used 10-fold cross-validation with internal sampling (see S1 File for details) and estimated the resulting ROC AUC (a measure equivalent to the C-statistic [14] used in other studies). The process was repeated 50 times to obtain the distribution of AUC scores. We compared the mean performance of our models with two extant methods, Patel et al. [8] and Basu et al. [9]. We evaluated the difference between the AUC scores using a simple t-test. For comparison with the method of Basu et al. we used a two-sample t-test as we have the equivalent distribution of AUC scores of their method. For Patel et al. we used the simple one-sample t-test as we only had the mean performance (without the variance across the cross-validation folds). Comparison was for prediction of CV events only, since the other two methods predicted SAE and not kidney-related events. To evaluate the quality of predicting kidney-related outcomes we compared our models with a univariate model that utilizes the treatment arm only (analogous to the original SPRINT study).

### Treatment recommendation—A simulation

How can our approach help the physician decide on aggressive vs. standard treatment given the risks for CV and adverse events? This decision is not trivial since aggressive treatment may decrease CV risk but induce higher risk for adverse events and vice versa [2,3]. One way to overcome this difficulty is to use both risks when deciding on the right treatment. To demonstrate this approach, we developed a method that recommends aggressive treatment to subjects with high risk for CV events but also keeps the AKI risk low. We studied this problem when the adverse event was AKI and did not include CKD as it was not defined for all SPRINT patients). We trained a logistic regression model using the predicted CV and AKI risks as covariates. A threshold value θ is set, and subjects with regression value above θ were assigned to the intensive treatment (in that sense, θ weighs the relative severity of CV vs. AKI events). See S1 File for more details.

### External validation on ACCORD data

We validated our models on the independent ACCORD dataset [15]. The ACCORD trial had similar goals to SPRINT but focused exclusively on type-2 diabetes mellitus patients. ACCORD was different from SPRINT in three additional aspects that are relevant for our analysis: (1) the set of CV events that defined a primary CV outcome. We addressed this by aggregating the same secondary CV outcomes of ACCORD as described by SPRINT (non fatal MI, non fatal stoke, coronary heart disease, heart failure and death from CV cause) to construct a matched composition for a primary CV outcome, (2) Since the definitions of kidney events were different in ACCORD, we validated only the CV models. (3) Finally, as the ACCORD trial did not report the 10-year Framingham Risk score it was excluded from this analysis.

In order to validate our CV models on the ACCORD data, we derived baseline and longitudinal models trained using internal sampling (see S1 File) from the entire SPRINT dataset, recalibrated them by adjusting the intercepts in order to match the overall event rates in ACCORD [14] and used them to predict CV events for all ACCORD participants. As in the SPRINT validation, patients with less than three BP measurements up to **t** months prior to the event/censoring were excluded from the prediction by the longitudinal model (n = 233 (4.9%), 346 (7.3%) for **t** = 6 and **t** = 12 respectively).

## Results

**Fig 1:**
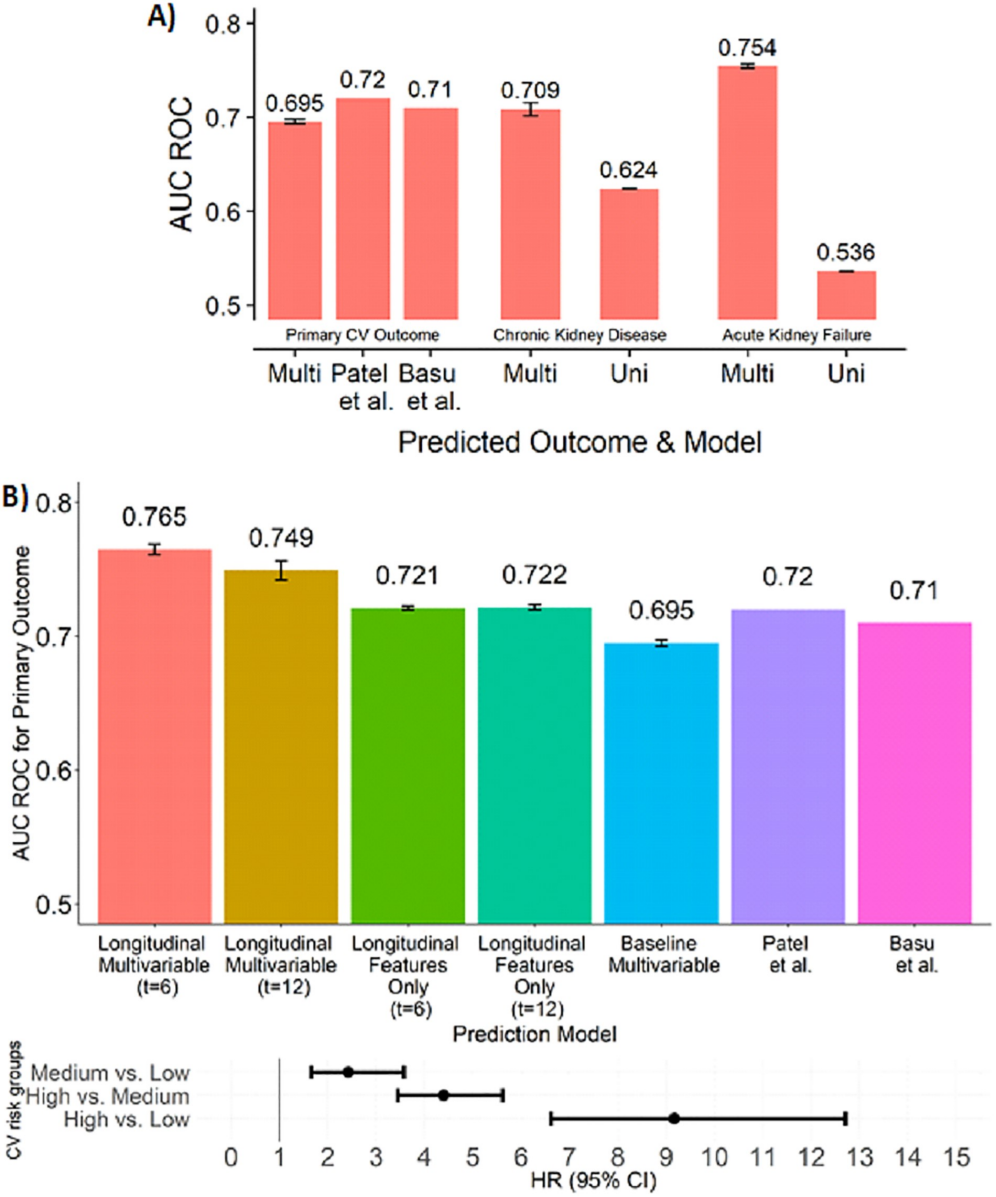
Performance of the predictors on the SPRINT cohort. A) Performance of baseline predictors in survival analysis (Multi: Multivariable, Uni: Univariate). Results from Basu et al. [9] and Patel et al. [8] were included for CV prediction, but were not available for kidney events. B) Top: Performance of all models in prediction of primary CV outcome (Longitudinal Features Only: a predictive model that utilized the five longitudinal features exclusively, see S1 Table). In A and B results are for 10-fold cross-validation repeated 50 times, with mean and standard errors. Note that sample sizes varied between outcomes in A and between t = 6 and t = 12 in B due to excluded samples. Bottom: Hazard ratio (HR) for different risk groups of SPRINT patients according to the longitudinal model (using t = 6). Patients without sufficient follow-up data (less than three BP measurements up to 6 months prior to censoring/event, n = 435) were excluded. Patients were divided into three equally sized risk groups (high, medium, low) according to their values as predicted by our model, and HR’s were calculated between the groups. HR between the high and low risk groups: 9.171 (6.617–12.710 95% CI); high vs. medium: 4.400 (3.450–5.611 95% CI); medium vs. low: 2.429 (1.653–3.571 95% CI, all HR P-values < 2*10^-16).

The results using only baseline variables are shown in Fig 1A. For all outcomes, multivariable prediction was far better (the univariate prediction using the treatment arm variate achieved 0.52 ROC AUC for CV outcome). This is expected, since multivariable methods leverage more information about the background risk of an individual. Multivariable prediction of primary CV outcome was slightly lower than for the models suggested in [8,9]. The advantage of the Basu and Patel models probably stems from the fact that they utilized multiple nonlinear combinations of variables, whereas our models used only two interactions between variables.

Fig 1B shows that our longitudinal model predicts the probability of the primary CV outcome better: Using the longitudinal data up to t = 6 and t = 12 months prior to a primary CV outcome event, we obtained a marked improvement compared to the multivariable baseline analysis and outperformed the models in [8,9] (p<2.4E-30, Student’s t-test, p<0.01 using Chebyshev’s inequality and avoiding the normal assumption). We emphasize that the primary outcome predicted was identical for all methods. Reassuringly, when ranking subjects by our predicted CV probability, we achieved hazard ratio of 9.171 between the first and third tertile of the cohort (Fig 1B). All our models showed good calibration when plotting the observed vs. predicted risk percentiles with modest overestimation on the top risk deciles (S2–S6 Figs). Notably, the most important features for the prediction of CV outcomes were the summary statistics extracted from longitudinal data and prior CV events (S3 Table). For predicting kidney-related outcomes, the most important features included known kidney markers such as urinary albumin to creatinine ratio, along with other general risk factors like the pulse pressure. For a full list of important features and their coefficients see S3–S5 Tables. The longitudinal model did not improve prediction of kidney related outcomes.

**Fig 2:**
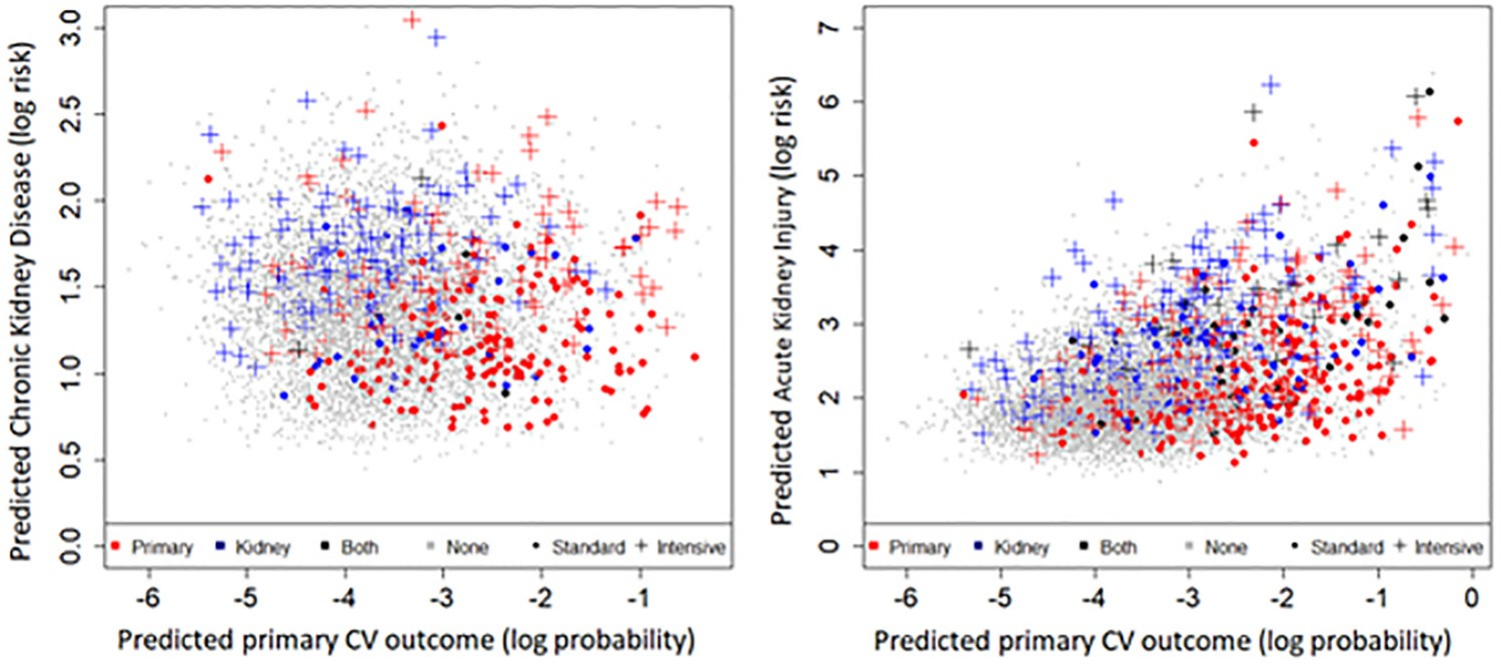
Predictions of the model on all SPRINT participants. Each spot shows a person’s predictions according to the final longitudinal model with **t** = 6. X axis: Primary CV outcome. Y axis: CKD or AKI. The point shape and color indicate the events that actually happened and the treatment arm in the SPRINT study for the individual.

Fig 2 shows the estimated risks of CV, CKD and AKI outcomes for all included individuals according to our final models. These plots give an overview of the distribution of risks and event probabilities calculated for the study patients. Both plots show a reasonable separation between the study arms. More importantly, they illustrate that actual events tend to cluster in areas where our models predict greater likelihood for an event (i.e., higher x coordinate values for CV outcomes and higher y values for kidney events).

### Treatment recommendation

We applied our recommendation system (see Methods) in a simulated scenario of assignment of SPRINT participants, based only on their pre-trial data. Our method partitioned the participants into two groups: Those with recommended intensive treatment (RI) and those with recommended standard treatment (RS). The RI group contained 4,246 patients (45.3%, S6 Table). Notably, the hazard ratio for CV events between RI and RS patients was 1.341 (1.136–1.583 95% CI, p-value = 0.002). Hence, patients for whom our system recommended intensive treatment were indeed at higher risk for CV events than patients who were recommended standard treatment. Moreover, the hazard ratio for AKI events between RI and RS patients was 0.676 (0.539–0.848 95% CI, p-value = 0.018). In other words, RI patients were at lower risk for AKI. This clearly demonstrates the ability of our recommendation system to identify patients who would benefit from intensive treatment and are less likely to experience AKI. A possible explanation for the success of our system could have been that the RI group is biased, as it includes more patients who were truly given intensive treatment. We ruled out this possibility by showing that the recommendations were not biased towards any treatment arm of the SPRINT study (S7 Table).

This analysis suggests that our models have the power to recommend treatment with reduced risks for both CV and AKI to the specific patient. Assigning treatment according to multiple variables is non-trivial, especially when multiple risks must be weighed, and the exact weight of each risk cannot be easily assessed. In our case, the choice of the threshold θ indirectly reflects the relative weights of each risk (see S10 Fig). While in our implementation we chose θ based on the training group (S1 File), one can modify θ to reflect personal severity assumptions. This analysis is retrospective and additional trials are needed in order to evaluate its accuracy and efficiency.

### External validation on ACCORD data

**Fig 3:**
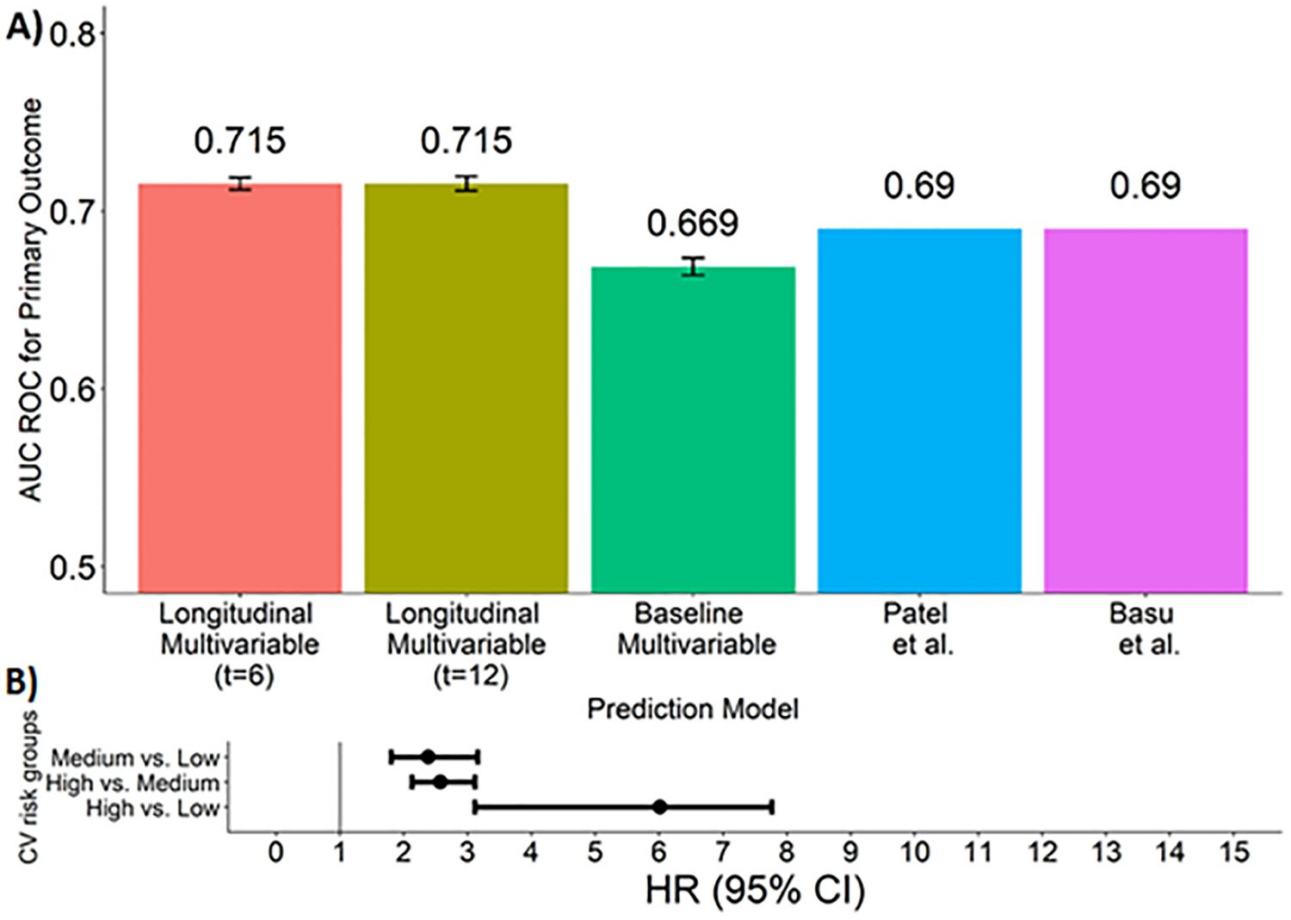
Performance of the predictors on an independent cohort. A) Performance of our models and other published models in prediction of primary CV outcome for patients from the ACCORD trial. All models were trained on the SPRINT data. For our models, mean and standard errors of 50 repetitions of the internal sampling are shown (see S1 File). As in Fig 1, sample sizes vary between the baseline and longitudinal models due to excluded samples. B) Hazard ratios for different risk groups of ACCORD patients. All ACCORD patients were divided into three equally sized risk groups and HR’s were calculated between the groups as explained for Fig 1B. HR between the high and low risk groups: 6.010 (3.110–7.763 95% CI); between high and medium: 2.573 (2.129–3.110 95% CI); between medium and low risk groups: 2.384 (1.801–3.157 95% CI). All HR P-values < 5.5*10^-9.

Fig 3A shows the performance of our SPRINT-derived longitudinal models in predicting CV events on the independent cohort of the ACCORD study. Performance was high, with only a modest decrease as compared to the SPRINT validation. Decent calibration was achieved when plotting the observed vs. predicted values for CV risk percentiles (S7–S9 Figs). Notably, our longitudinal models outperformed the models in [8,9] for predicting CV events on the ACCORD data (p<7.7E-37, Student’s t-test, p<0.01 using Chebyshev’s inequality and avoiding the normal assumption). Again, we stress that the predicted primary outcome was the same for all methods. When ranking the ACCORD patients by their predicted risk, the hazard ratio between top and bottom tertile was 5.999 (4.65–7.73995% CI, P-value < 5.5*10^-9) (Fig 3B).

## Discussion

Our analysis highlights the value of longitudinal treatment response data for accurate prediction of CV events. Such analysis incorporates variables measured later than the baseline variables and reflects the treatment response. Thus, they offer direct information not used by extant models. While seemingly trivial, integration of longitudinal data into such prediction models has proven to be challenging [16]. To the best of our knowledge, our study is the first to leverage longitudinal blood pressure measurements to significantly improve CV risk prediction. By quantifying simultaneously the CV benefit and the kidney-related risks, our predictions can assist clinicians in treatment decisions. We demonstrated such usage here by developing a tool that predicts both risks for a patient considered for aggressive treatment. We show that it successfully discriminates between patients who would benefit from such treatment and those who are more likely to be harmed by it.

The SPRINT randomized controlled trial (RCT) showed a significant effect of aggressive treatment for hypertension on CV events, but observed that the treatment also increased the risk of renal complications. It is important to note that the main goal of an RCT, by definition, is to provide an estimate for one causal factor. In other words, the randomization is used to separate the effect of the treatment from other factors such that the causal effect of the treatment on the outcome would be identifiable [17]. However, the task of predicting the risk for a specific subject, the focus of precision medicine, is fundamentally different from the goal of an RCT. Such prediction should consider as many presumed causal risk factors (or at least proxies of these factors) as possible when making the assessment. Naturally, once the SPRINT RCT showed the significance of the treatment, this variable should be added into the model, as was recapitulated in our results. It is therefore not surprising that our analysis and those of others easily improved upon a univariate model akin to that used in the SPRINT study.

In this study, we adopted a retrospective study design that utilized data from the SPRINT trial to train powerful predictive models that rely on both baseline and longitudinal follow-up data, for personalized prognosis of cardiovascular patients. While our analysis is limited due to the retrospective nature of the data, our careful validations illustrate the usefulness of integrating regularly collected longitudinal data into prediction models. Recently, Pool et al.[16] examined whether incorporating cumulative SBP measures that summarize SBP levels collected over time improve atherosclerotic cardiovascular disease prediction. Their results show only modest improvement by incorporating cumulative SBP in the prediction as compared to a single SBP measure. In contrast, we presented a marked improvement by introducing novel features derived from the longitudinal data. Possible explanations of our better result is the fact that we make a more complex use of the response to treatment. Notably, our results show a clear improvement trend in prediction quality: multivariable models outperform univariate models, and multivariable models that use baseline and longitudinal features improve over models that incorporate baseline data only. Taken together, our main contribution is illustrating how longitudinal data can contribute to more precise risk estimation.

Our models performed well in internal validation on SPRINT dataset. The validation on the independent ACCORD dataset further confirmed the usefulness and generalizability of the CV outcome predictor. In an additional evaluation of the model, we tested it in cross validation on each treatment arm of the SPRINT study separately, in order to rule out a possible concern that the treatment assignment significantly confounded our results. The results (S11 Fig) when testing each group separately still outperform the baseline model, with a modest decrease in performance. Some decrease is expected, due to reduced sample size and exclusion of the treatment type feature from the model (an important feature for CV prediction, S3 Table). Nevertheless, this experiment demonstrated that inclusion of longitudinal data in the model robustly improved predictive power.

Several other machine learning studies utilized data from SPRINT and demonstrated the advantage of multivariable predictors [8,9]. Our study is the first to utilize the longitudinal data and show that using these data significantly improves risk prediction. Our longitudinal models clearly illustrate that blood pressure measurements taken well ahead of the primary outcome contribute to prediction accuracy. This observation was corroborated in our results in two ways: 1) features of the longitudinal data had higher importance in our models than the baseline characteristics, and 2) longitudinal models that use only BP measurements outperformed all baseline models, each of which utilizing 15–20 features (Fig 1B). More generally, our results suggest that longitudinal data, which is expected to be available more broadly and at denser sampling frequency owing to new technologies (e.g. wearable devices), should become a standard tool for assessing and improving treatment policies. The advantage of using the longitudinal data is probably in part because the data carry information about the response to the treatment, which is a downstream effect of the treatment arm and a more direct risk factor.

Our study has some major limitations. First, we carried out a retrospective analysis. A randomized trial is needed to precisely estimate the effect of the new recommendation system and rule out post-randomization biases in the predictors. Second, as explained above, we decided to consider only kidney related SAEs, because of the results of the SPRINT study. Larger sample sizes in future studies may enable other predictions. In prediction of all SAE, extant models outperformed our baseline model (S12 Fig). Third, individual patient adherence to the assigned treatments may have introduced bias to our results. The fact that we cannot control for such a confounding effect is a limitation of this analysis. Finally, our main external validation was performed using the ACCORD dataset. Our results clearly showed the generalizability of our model, but the exact estimates of the hazard ratios should be interpreted with caution, as the ACCORD trial focused on diabetes subjects. In addition, since both SPRINT and ACCORD contained exclusively patients from the USA, additional validation on cohorts from other countries is needed to demonstrate the robustness of the predictors.

R scripts for reproducing all our results are available from https://github.com/Shamir-Lab/SPRINT

## Supporting information

S1 File Supplementary methods. (DOCX)

S1 Fig Utilizing the SPRINT longitudinal data in a personalized predictor. Features were derived from blood pressure measurements taken in periodic clinic visits. Measurements within t = 6 or 12 months prior to the event (or to follow-up end) were excluded in order to avoid information leakage.
(TIF)

S2 Fig CV baseline model calibration curve on SPRINT validation. Intercept: 0.0001, slope: 0.768, R2: 0.985.
(TIF)

S3 Fig AKI baseline model calibration curve on SPRINT validation. Intercept: 0.002, slope: 0.628, R2: 0.973.
(TIF)

S4 Fig CKD baseline model calibration curve on SPRINT validation. Intercept: 0.002, slope: 0.703, R2: 0.943.
(TIF)

S5 Fig CV longitudinal model (t = 6) calibration curve on SPRINT validation. Intercept: 0.002, Slope: 0.85, R2: 0.992.
(TIF)

S6 Fig CV longitudinal model (t = 12) calibration curve on SPRINT validation. Intercept: 0.007, Slope: 0.660, R2: 0.973.
(TIF)

S7 Fig CV baseline model calibration curve on external validation using ACCORD. Intercept: -0.021, Slope: 1.059, R2: 0.944.
(TIF)

S8 Fig CV longitudinal model (t = 6) calibration curve on external validation using ACCORD. Intercept: 0.036, Slope: 0.715, R2: 0.984.
(TIF)

S9 Fig CV longitudinal model (t = 12) calibration on external validation using ACCORD. Intercept: 0.032, Slope: 0.702, R2: 0.964.
(TIF)

S10 Fig The effect of the threshold θ on the results of the treatment recommendation system. The colored points show the HR for CV and AKI in the RI vs. RS groups as a function of θ. The recommendation system must find a value θ that maximizes the HR for CV while keeping the HR for AKI low (see Methods). This is represented in the graph as points on the X axis where the red dot is above 1 and the blue dot is below 1 (e.g. for θ ∈ [0.51,0.57]). The figure demonstrates the treatment decision tension described in **Methods**: as θ grows, the HR for CV increases, raising the need for intensive treatment. However, the HR for AKI also increases with θ, making the patients in RI more vulnerable for AKI. Therefore, we need to find θ that balances the two: assigning the patients at higher CV risk to RI without compromising them with high risk for AKI. The black dots specify the fraction of patients in RI as a function of θ. Note that when θ ≤ 0.4 or ≥ 0.6, the vast majority of patients are assigned to one of the groups and the computed HR values are unstable due to the extreme imbalance. (For the sake of the presentation here, results were computed for the entire cohort. In the pipeline described in S1 File we chose a different θ at every fold according to the training group).
(TIF)

S11 Fig Performance of CV event prediction by the longitudinal model (t = 6) when the model is tested in 10-fold cross validation using the entire SPRINT cohort ("All patients"), the group of patients that received intensive treatment in SPRINT ("Intensive") only, or the group of patients that received standard treatment in SPRINT ("Standard") only. (TIF)

S12 Fig Predicting general SAE. Our baseline multivariate model for prediction of a general SAE does not improve upon the results of extant models. Results for our model are mean and standard error for 50 repeats.
(TIF)

S1 Table Mean static and dynamic characteristics of SPRINT study participants; ± SD. All baseline features were used as candidate predictors for all models (baseline and longitudinal), dynamic features were included in the longitudinal models only. The last five characteristics were extracted from longitudinal data, i.e., post-randomization. The last three are features of the linear curves fitted for the series of longitudinal BP values of each individual using t = 6. The static characteristics are as reported in the original SPRINT study, with the exception of the Farmingham score, which was corrected.
(PDF)

S2 Table The relevant results of the SPRINT study. Hazard ratio p-value < 0.001 for all outcomes.
(PDF)

S3 Table Feature importance for predicting Primary CV outcome by the longitudinal model (using t = 6, logistic regression). Bold: summary statistics extracted from longitudinal data. Mean coefficients and SD are calculated using 10000 repeats. Positive mean coefficient implies positive risk for CV event.
(PDF)

S4 Table Feature importance for predicting acute kidney injury or renal failure by the baseline model (CoxPH). Mean coefficients and SD are calculated using 10000 iterations. Mean coefficient > 1implies positive risk effect for CV event.
(PDF)

S5 Table Feature importance for predicting novel Chronic Kidney Disease (CKD) for non-CKD patients at baseline by the baseline model (CoxPH). Mean coefficients and SD are calculated using 1000 iterations. Mean coefficient > 1 implies positive risk effect for CV event.
(PDF)

S6 Table Hazard ratios for tested outcomes based on the recommended assignments of our method. Our assignment shows significant increase in CV hazard for the group of patients that would have been recommended intensive treatment along with significant decrease in hazard for AKI.
(PDF)

S7 Table Breakdown of patient assignment to treatment arms by the SPRINT study and by our recommendation system. The recommended assignment of patients to the two arms is not biased towards any of the original arms.
(PDF)
