# Supplementary material for: Personalized prediction of adverse heart and kidney events using baseline and longitudinal data from SPRINT and ACCORD
Source: PLoS One. 2019 Aug 8;14(8):e0219728. doi: 10.1371/journal.pone.0219728 (PMC6687091; doi:10.1371/journal.pone.0219728)
Supplement: S1 File — (DOCX) [file pone.0219728.s001.docx]

**Supplementary Methods**

Internal sampling: We used CoxPH [1] regression and logistic regression (LR) [2,3] as predictors in the baseline and longitudinal models, respectfully. Training a predictor when the labels in the training dataset are unbalanced (e.g. predicting a CV outcome when the overwhelming majority of patients in the training data did not experience a CV outcome) is a challenge, because the derived predictor might underestimate the risk due to such imbalance. To overcome this difficulty, we used an internal sampling method [4] on top of 10-fold cross validation as follows. Samples were split into 10 equal size sets, and in each iteration (fold) one set served as the test set and the remaining samples were used for training. In each iteration, if *n* subjects in the training set were positive for the event, we sampled from that set *n* additional subjects that were negative for the event and trained the model on these *2n* patients. We repeated this process ten times in each iteration, deriving a different predictor each time. The final predicted value of a patient in the test set was the mean of the values computed for the patient by each of the ten derived predictors. This internal sampling method was highly beneficial in the longitudinal model, but showed no advantage in the baseline models. For this reason, we used LR with internal sampling on the longitudinal data and CoxPH regression without sampling on the baseline data.

Recommendation method pipeline: 1) Using the baseline CoxPH predictors, CV risk assuming standard treatment and AKI risk assuming intensive treatment were predicted for each patient. These scores were predicted using the baseline data only, mimicking a real life situation where a physician needs to decide on a treatment for the first time. 2) A final logistic regression model was derived based on the two risks as regressors. In the final model, we only used subjects that had either CV or AKI events for training, so that low predicted regression value corresponds to high AKI risk and vice versa (patients who had both CV and AKI were labeled with CV only). The recommendation rule was RI for the patients with regression value > θ and RS for the rest. In order to choose the optimal θ based on the training group, we screened a range of θ values; for each θ we assigned the patients from the training group with regression value > θ into $RI_{\theta}$ and the rest to $RS_{\theta}$, and computed the HR for CV between $RI_{\theta}$ and $RS_{\theta}$. The final θ was chosen as the value that maximized the HR among all θ's for which the HR for AKI was ≤ 1. 3) In every cross-validation iteration $\theta$ was chosen according to the training group, the patients from the test group with predicted value > $\theta$ were assigned to RI, and the rest were assigned to RS. The method was tested in 10-fold cross validation with internal sampling (see **Supplementary Methods** for details) by repeatedly holding out 10% of the samples as a test set, computing the final model and the optimal $\theta$ using data from the other participants and assigning to RI subjects from the test set that had predicted value > θ. To avoid unstable assignments of patients to treatment groups, we ran this pipeline for 101 rounds, with random 10-fold split and cross validation in each round, deriving a different assignment in each round for every patient. The final assignment for each patient was set by majority vote (i.e., the patient was assigned to RI if and only if it was assigned to RI in more than 50 rounds).

**Supplemental References**

1. Cox DR. Regression models and life‐tables. J R Stat Soc Ser B. Wiley Online Library; 1972;34: 187–202.

2. Cox DR. The regression analysis of binary sequences. J R Stat Soc Ser B. Wiley Online Library; 1958;20: 215–232.

3. Hastie T, Tibshirani R, Friedman J, Franklin J. The elements of statistical learning: data mining, inference and prediction. Math Intell. Springer; 2005;27: 83–85.

4. Amar D, Izraeli S, Shamir R. Utilizing somatic mutation data from numerous studies for cancer research: proof of concept and applications. Oncogene. Nature Publishing Group; 2017;36: 3375.
