# Supplementary figures and images for: Personalized prediction of adverse heart and kidney events using baseline and longitudinal data from SPRINT and ACCORD
Source: PLoS One. 2019 Aug 8;14(8):e0219728. doi: 10.1371/journal.pone.0219728 (PMC6687091; doi:10.1371/journal.pone.0219728)

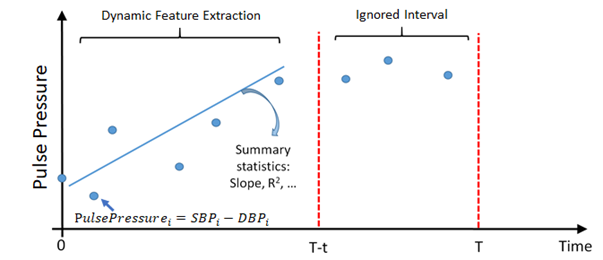

Supplement: S1 Fig — Features were derived from blood pressure measurements taken in periodic clinic visits. Measurements within t = 6 or 12 months prior to the event (or to follow-up end) were excluded in order to avoid information leakage. (TIF) [file pone.0219728.s002.tif]

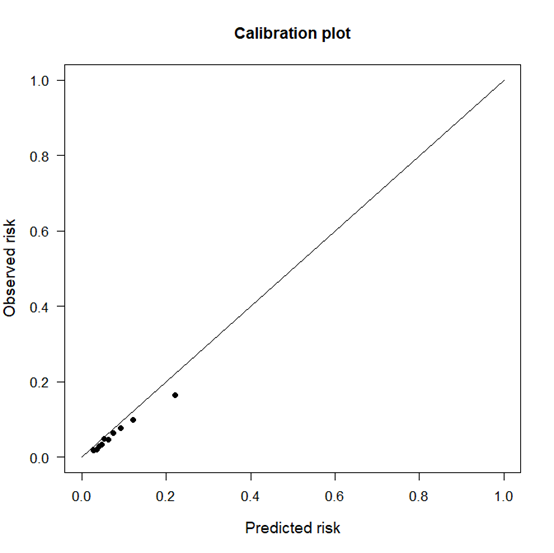

Supplement: S2 Fig — Intercept: 0.0001, slope: 0.768, R2: 0.985. (TIF) [file pone.0219728.s003.tif]

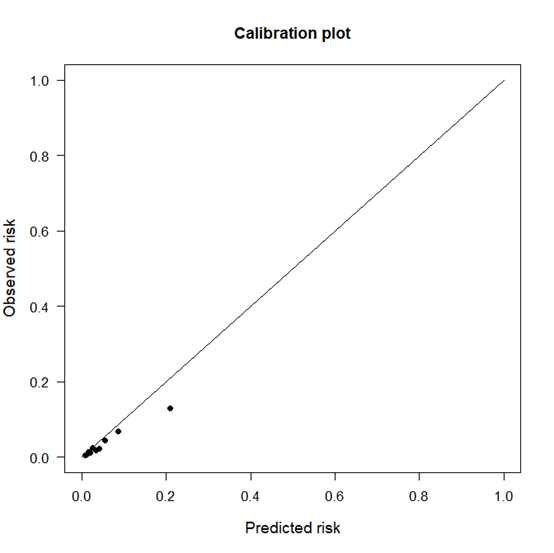

Supplement: S3 Fig — Intercept: 0.002, slope: 0.628, R2: 0.973. (TIF) [file pone.0219728.s004.tif]

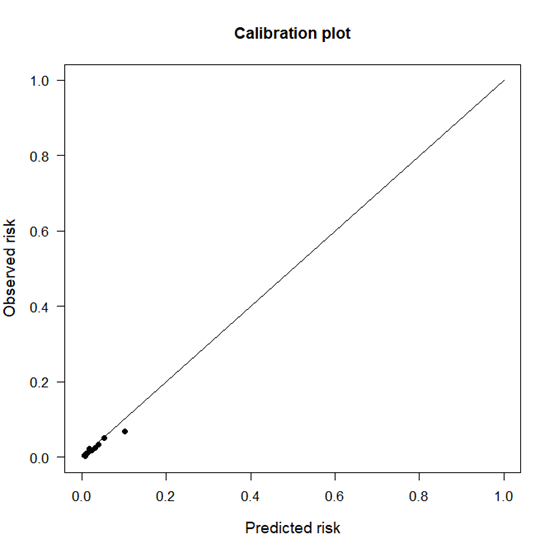

Supplement: S4 Fig — Intercept: 0.002, slope: 0.703, R2: 0.943. (TIF) [file pone.0219728.s005.tif]

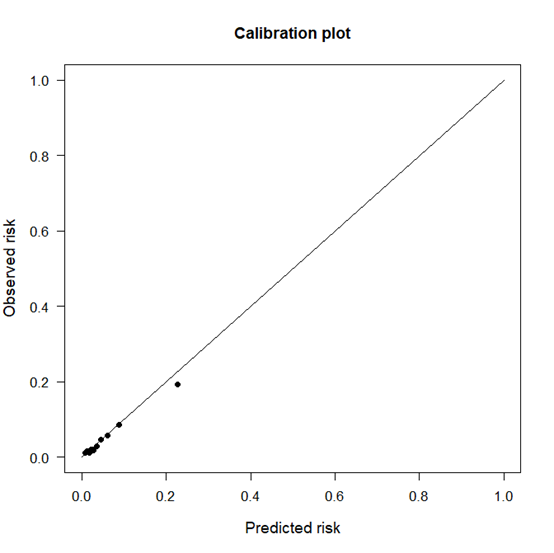

Supplement: S5 Fig — Intercept: 0.002, Slope: 0.85, R2: 0.992. (TIF) [file pone.0219728.s006.tif]

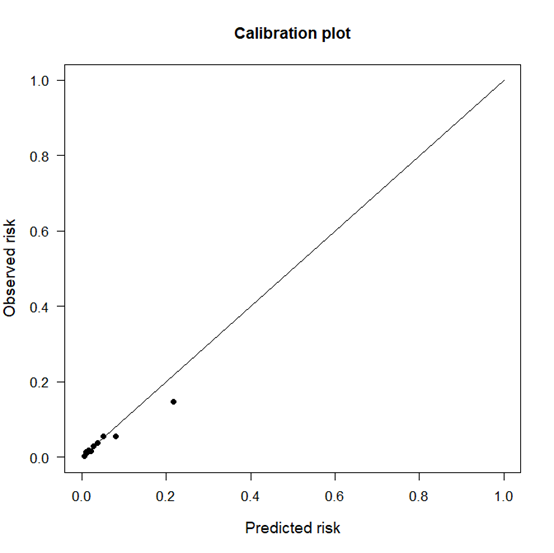

Supplement: S6 Fig — Intercept: 0.007, Slope: 0.660, R2: 0.973. (TIF) [file pone.0219728.s007.tif]

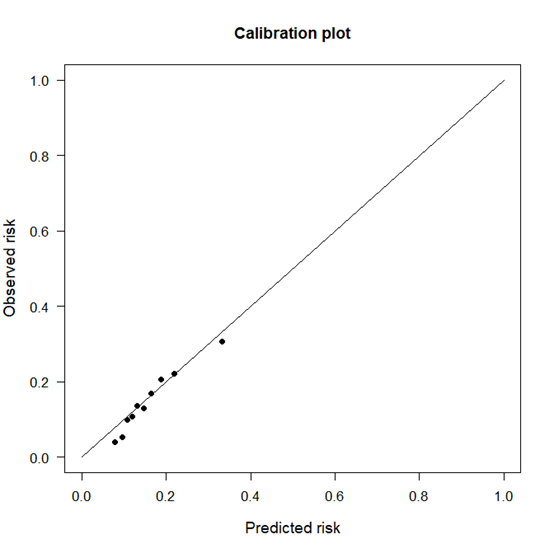

Supplement: S7 Fig — Intercept: -0.021, Slope: 1.059, R2: 0.944. (TIF) [file pone.0219728.s008.tif]

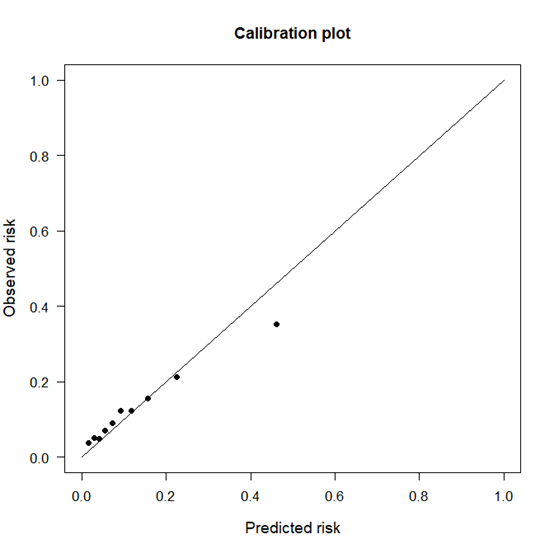

Supplement: S8 Fig — Intercept: 0.036, Slope: 0.715, R2: 0.984. (TIF) [file pone.0219728.s009.tif]

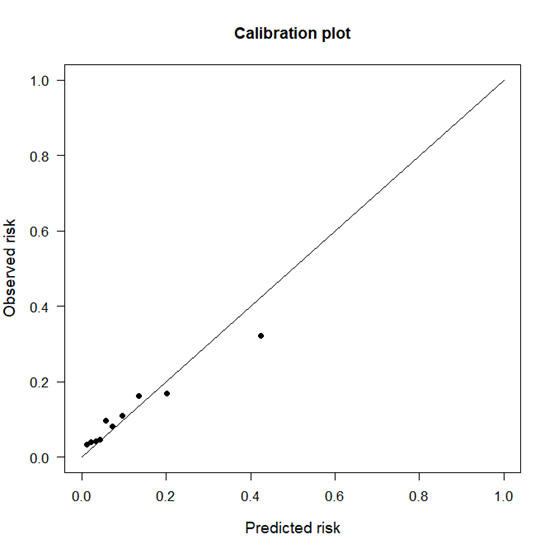

Supplement: S9 Fig — Intercept: 0.032, Slope: 0.702, R2: 0.964. (TIF) [file pone.0219728.s010.tif]

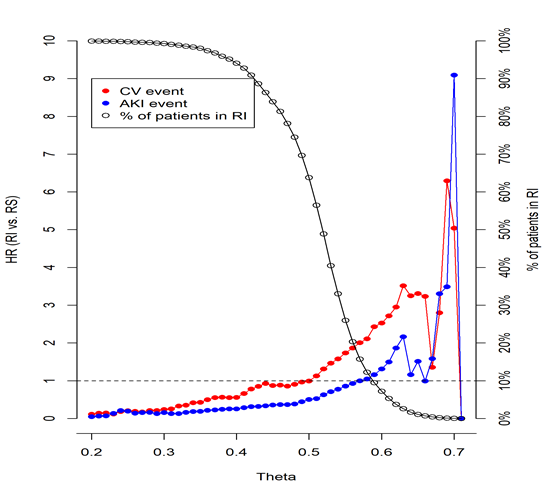

Supplement: S10 Fig — The colored points show the HR for CV and AKI in the RI vs. RS groups as a function of θ. The recommendation system must find a value θ that maximizes the HR for CV while keeping the HR for AKI low (see Methods). This is represented in the graph as points on the X axis where the red dot is above 1 and the blue dot is below 1 (e.g. for θ ∈ [0.51,0.57]). The figure demonstrates the treatment decision tension described in Methods: as θ grows, the HR for CV increases, raising the need for intensive treatment. However, the HR for AKI also increases with θ, making the patients in RI more vulnerable for AKI. Therefore, we need to find θ that balances the two: assigning the patients at higher CV risk to RI without compromising them with high risk for AKI. The black dots specify the fraction of patients in RI as a function of θ. Note that when θ ≤ 0.4 or ≥ 0.6, the vast majority of patients are assigned to one of the groups and the computed HR values are unstable due to the extreme imbalance. (For the sake of the presentation here, results were computed for the entire cohort. In the pipeline described in S1 File we chose a different θ at every fold according to the training group). (TIF) [file pone.0219728.s011.tif]

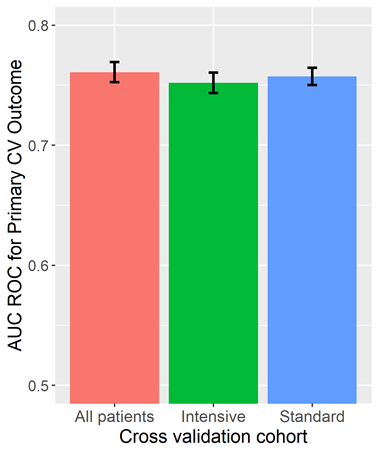

Supplement: S11 Fig — (TIF) [file pone.0219728.s012.tif]

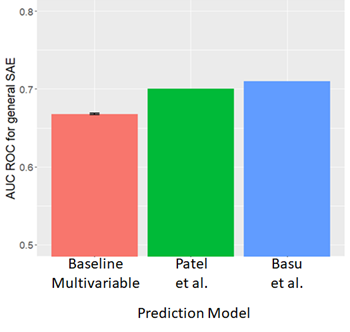

Supplement: S12 Fig — Our baseline multivariate model for prediction of a general SAE does not improve upon the results of extant models. Results for our model are mean and standard error for 50 repeats. (TIF) [file pone.0219728.s013.tif]
