# Supplementary material for: Personalized prediction of adverse heart and kidney events using baseline and longitudinal data from SPRINT and ACCORD
Source: PLoS One. 2019 Aug 8;14(8):e0219728. doi: 10.1371/journal.pone.0219728 (PMC6687091; doi:10.1371/journal.pone.0219728)
Supplement: S1 Table — All baseline features were used as candidate predictors for all models (baseline and longitudinal), dynamic features were included in the longitudinal models only. The last five characteristics were extracted from longitudinal data, i.e., post-randomization. The last three are features of the linear curves fitted for the series of longitudinal BP values of each individual using t = 6. The static characteristics are as reported in the original SPRINT study, with the exception of the Farmingham score, which was corrected. (PDF) [file pone.0219728.s014.pdf]

characteristic	Intensive Treatment (N = 4678)	Standard Treatment (N = 4683)
**Baseline (static)**		
Age	67.9±9.4	67.9±9.5
Female Sex no. (%)	1684 (36.0)	1648 (35.2)
Ethnic black no. (%)	1454 (31.1)	1493 (31.9)
Framingham Score (corrected)	24.81±12.62	24.81±12.45
Past CV Event no. (%)	940 (20.1)	937 (20.0)
Chronic Kidney Disease no. (%)	1330 (28.4)	1316 (28.1)
Systolic Blood Pressure- mmHg	139.7±15.8	139.7±15.4
Diastolic Blood Pressure- mmHg	78.2±11.9	78.0±12.0
Serum Creatinine- mg/dl	1.07±0.34	1.08±0.34
Estimated GFR (all participants)- ml/min/1.73 m^2	71.8±20.7	71.7±20.5
Fasting total cholesterol- mg/dl	190.2±41.4	190.0±40.9
Fasting HDL cholesterol- mg/dl	52.9±14.3	52.8±14.6
Fasting total triglycerides- mg/dl	124.8±85.8	127.1±95.0
Fasting plasma glucose- mg/dl	98.8±13.7	98.8±13.4
Statin use no. (%)	1978/4645 (42.6)	2076/4640 (44.7)
Aspirin use no. (%)	2406/4661 (51.6)	2350/4666 (50.4)
Current smoker no. (%)	639 (13.7)	601 (12.8)
Former smoker no. (%)	1977 (42.2)	1996 (42.6)
BMI	29.9±5.8	29.8±5.7
Antihypertensive agents- no./patient	1.8±1	1.8±1
Ratio of urinary albumin (mg) to creatinine (g)	44.1±178.7	41.1±152.9
**Longitudinal (dynamic)**		

Mean pulse pressure	54.25±10.37	60.03±10.48
Difference between maximal and minimal measured BP values	25.84±10.63	26.40±10.35
F statistic p-value for the overall significance of the linear model	0.38±0.30	0.42±0.30
R2 for correlation between the measured BP values and those predicted by the linear model	0.16±0.18	0.13±0.17
Slope of linear model	-0.18±0.92	0.01±0.91

S1 Table. Mean static and dynamic characteristics of SPRINT study participants; ± SD. All baseline features were used as candidate predictors for all models (baseline and longitudinal), dynamic features were included in the longitudinal models only. The last five characteristics were extracted from longitudinal data, i.e., post-randomization. The last three are features of the linear curves fitted for the series of longitudinal BP values of each individual using t=6. The static characteristics are as reported in the original SPRINT study, with the exception of the Farmingham score, which was corrected [1].

[1] Warner F, Dhruva SS, Ross JS, Dey P, Murugiah K, Krumholz HM. Correction of the Framingham Risk Score Data Reported in SPRINT. bioRxiv. 2017; 1–5. doi:10.1101/235358
