# Supplementary material for: Personalized prediction of adverse heart and kidney events using baseline and longitudinal data from SPRINT and ACCORD
Source: PLoS One. 2019 Aug 8;14(8):e0219728. doi: 10.1371/journal.pone.0219728 (PMC6687091; doi:10.1371/journal.pone.0219728)
Supplement: S2 Table — Hazard ratio p-value < 0.001 for all outcomes. (PDF) [file pone.0219728.s015.pdf]

Outcome	Intensive Treatment (N = 4678)	Standard Treatment (N = 4683)	Hazard Ratio (95% CI)
Primary CV outcome no. (%)	243 (5.2)	319 (6.8)	0.75 (0.64-0.89)
Acute Renal Injury or Renal Failure no. (%)	204 (4.4)	120 (2.6)	1.71
	Participants without Chronic Kidney Disease (CKD) at baseline		
	Intensive Treatment (N = 3332)	Standard Treatment (N = 3345)	
Novel CKD no. (%)	127 (3.8)	37 (1.1)	3.49 (2.44-5.10)

S2 Table. The relevant results of the SPRINT study. Hazard ratio p-value < 0.001 for all outcomes.
