# Supplementary material for: Personalized prediction of adverse heart and kidney events using baseline and longitudinal data from SPRINT and ACCORD
Source: PLoS One. 2019 Aug 8;14(8):e0219728. doi: 10.1371/journal.pone.0219728 (PMC6687091; doi:10.1371/journal.pone.0219728)
Supplement: S3 Table — Bold: summary statistics extracted from longitudinal data. Mean coefficients and SD are calculated using 10000 repeats. Positive mean coefficient implies positive risk for CV event. (PDF) [file pone.0219728.s016.pdf]

Characteristic	Percent of sampling runs in which the characteristic appeared in the final model (N=10000)	Mean	SD
**F statistic p-value for the overall significance of the linear model**	100	2.153	0.438
**R^2 for correlation between the measured BP values and those predicted by the linear model**	100	4.094	0.850
**Difference between maximal and minimal measured BP values**	100	-0.037	0.009
Past Clinical CV event	96.6	1.049	0.374
Intensive treatment	96.03	-0.413	0.173
Number of hypertensive agents	94.17	0.156	0.081
Ratio of urinary albumin to creatinine	94.11	0.001	0.0008
Framingham 10 years risk score	92.6	0.024	0.012
Age	90.12	0.027	0.015
Serum creatinine	89.99	0.74	0.477
**Slope of linear model**	89.02	0.003	0.098
Current smoker	87.46	0.413	0.280
Female sex	87.37	0.416	0.251
Former smoker	82.68	0.162	0.157
**Mean pulse pressure**	80.82	0.012	0.009

Statin usage	74.59	-0.035	0.165
BMI	72.93	0.001	0.014
Ethnic white	72.26	-0.289	0.258
Ethnic Hispanic	71.28	0.159	0.642
Chronic kidney disease	70.18	-0.023	0.237
Cholesterol	62.43	0.001	0.002
HDL/Cholesterol ratio	57.77	-1.125	1.24
EGFR/Serum creatinine ratio	56.72	0.003	0.002
Pulse pressure at baseline	54.17	-0.0009	0.007
HDL	47.67	-0.002	0.006
Ethnic black	30.68	0.078	0.178
EGFR	16.79	-0.003	0.003

S3 Table. Feature importance for predicting Primary CV outcome by the longitudinal model (using t=6, logistic regression). Bold: summary statistics extracted from longitudinal data. Mean coefficients and SD are calculated using 10000 repeats. Positive mean coefficient implies positive risk for CV event.
