# Supplementary material for: Personalized prediction of adverse heart and kidney events using baseline and longitudinal data from SPRINT and ACCORD
Source: PLoS One. 2019 Aug 8;14(8):e0219728. doi: 10.1371/journal.pone.0219728 (PMC6687091; doi:10.1371/journal.pone.0219728)
Supplement: S4 Table — Mean coefficients and SD are calculated using 10000 iterations. Mean coefficient > 1implies positive risk effect for CV event. (PDF) [file pone.0219728.s017.pdf]

Characteristic	Percent of sampling runs in which the characteristic appeared in the final model (N=10000)	Mean	SD
Serum creatinine	71.2	1.474	1.064
Ratio of urinary albumin to creatinine	71.05	0.0015	0.0022
Intensive treatment	69.91	0.604	0.631
Past subclinical CV event	60.59	-0.282	1.103
Former smoker	60.04	0.346	0.644
Pulse pressure at baseline	58.12	0.011	0.024
Number of hypertensive agents	58.01	0.149	0.306
BMI	57.40	0.016	0.065
Statin usage	56.85	-0.214	0.685
Chronic kidney disease	56.85	0.688	0.726
Current smoker	53.34	0.509	0.933
Framingham 10 years risk score	52.31	0.024	0.026
Ethnic white	49.52	-0.394	0.818
Past clinical CV event	49.39	0.643	0.777
Cholesterol	47.64	-0.003	0.009
Ethnic black	45.54	0.498	0.632
Female sex	42.77	-0.311	0.718
HDL to cholesterol ratio	40.45	2.971	4.204
Age	40.24	0.024	0.043

HDL	38.55	-0.0003	0.027
EGFR to serum creatinine ratio	30.41	-0.006	0.011
Past clinical CV event	27.10	0.160	0.844
Ethnic Hispanic	23.17	0.058	1.367
EGFR	18.99	-0.018	0.020

S4 Table. Feature importance for predicting acute kidney injury or renal failure by the baseline model (CoxPH). Mean coefficients and SD are calculated using 10000 iterations. Mean coefficient > 1implies positive risk effect for CV event.
