# Supplementary material for: Personalized prediction of adverse heart and kidney events using baseline and longitudinal data from SPRINT and ACCORD
Source: PLoS One. 2019 Aug 8;14(8):e0219728. doi: 10.1371/journal.pone.0219728 (PMC6687091; doi:10.1371/journal.pone.0219728)
Supplement: S5 Table — Mean coefficients and SD are calculated using 1000 iterations. Mean coefficient > 1 implies positive risk effect for CV event. (PDF) [file pone.0219728.s018.pdf]

Characteristic	Percent of sampling runs in which the characteristic occurred as a significant feature ($\beta \neq 0$: p-value <0.01, N=10000)	Mean	SD
Pulse pressure	100	1.035	0.002
Intensive treatment	100	3.316	0.204
Ratio of urinary albumin to creatinine	90.63	1.0006	0.0001
Female sex	18.3	2.096	0.560

S5 Table. Feature importance for predicting novel Chronic Kidney Disease (CKD) for non-CKD patients at baseline by the baseline model (CoxPH). Mean coefficients and SD are calculated using 1000 iterations. Mean coefficient > 1 implies positive risk effect for CV event.
