# Supplementary material for: Personalized prediction of adverse heart and kidney events using baseline and longitudinal data from SPRINT and ACCORD
Source: PLoS One. 2019 Aug 8;14(8):e0219728. doi: 10.1371/journal.pone.0219728 (PMC6687091; doi:10.1371/journal.pone.0219728)
Supplement: S6 Table — Our assignment shows significant increase in CV hazard for the group of patients that would have been recommended intensive treatment along with significant decrease in hazard for AKI. (PDF) [file pone.0219728.s019.pdf]

Outcome	Recommended Intensive (N=4246)	Recommended Standard (N=5115)	HR (95% CI)	P-value
CV events (%)	287 (0.067)	275 (0.053)	1.341(1.136-1.583)	0.002
AKI events (%)	124 (0.029)	200 (0.039)	0.676 (0.539-0.848)	0.018

S6 Table. Hazard ratios for tested outcomes based on the recommended assignments of our method. Our assignment shows significant increase in CV hazard for the group of patients that would have been recommended intensive treatment along with significant decrease in hazard for AKI.
