# Supplementary material for: Personalized prediction of adverse heart and kidney events using baseline and longitudinal data from SPRINT and ACCORD
Source: PLoS One. 2019 Aug 8;14(8):e0219728. doi: 10.1371/journal.pone.0219728 (PMC6687091; doi:10.1371/journal.pone.0219728)
Supplement: S7 Table — The recommended assignment of patients to the two arms is not biased towards any of the original arms. (PDF) [file pone.0219728.s020.pdf]

P-value for interaction: 0.3292		**Original assignment**	
		Intensive	Standard
**Recommended assignment**	Intensive	2098	2148
	standard	2580	2535

S7 Table. Breakdown of patient assignment to treatment arms by the SPRINT study and by our recommendation system. The recommended assignment of patients to the two arms is not biased towards any of the original arms.
